# Investigation of the differences between the medical personnel’s and general population’s view on the doctor-patient relationship in China by a cross-sectional survey

**DOI:** 10.1186/s12992-020-00625-9

**Published:** 2020-10-15

**Authors:** Tianqing Sang, Hongli Zhou, Muhan Li, Wenting Li, Haibo Shi, Haibin Chen, Hongguang Zhou

**Affiliations:** 1 Institute of Oncology, The First Clinical Medical College, Nanjing University of Chinese Medicine, Nanjing, 210046 Jiangsu Province China; 2 Liaoning University of Chinese Medicine, Shenyang, 110847 Liaoning Province China; 3 Wuxi Xishan Hospital of Traditional Chinese Medicine, Wuxi, 214000 Jiangsu Province China; 4 Science and Technology Department, Jiangsu Collaborative Innovation Center of Traditional Chinese Medicine Prevention and Treatment of Tumor, Nanjing University of Chinese Medicine, Nanjing, 210046 Jiangsu Province China; 5 Department of Oncology, Affiliated Hospital of Nanjing University of Chinese Medicine, Nanjing, 210029 Jiangsu Province China

**Keywords:** Doctor-patient relationship (DPR), Medical dispute, Patient satisfaction, People’s republic of China

## Abstract

**Background:**

Due to economic development and an increase in the aging population, the demand for medical resources is increasing. A good doctor-patient relationship (DPR) can optimize patients’ medical experience and improve treatment efficiency. The DPR, however, is currently in crisis in China. To explore ways to improve DPR, this study assessed the views on the status of the DPR, medical services, and the general situation of medical work among medical personnel (MP) and the general population (GP).

**Methods:**

This cross-sectional study, conducted between December 2019 and March 2020, targeted the MP and the GP in Nanjing City, Jiangsu Province, and Zhengzhou City, Henan Province. A total of 154 MP and 329 GP answered a self-administered questionnaire through Questionnaire Star and WeChat apps. Wilcoxon’s Sign Rank Test, Chi-square test, and frequency distributions and percentages were used to process the data.

**Results:**

Only 11.04% of the MP and 14.89% of the GP believed that the current DPR was harmonious. Moreover, 54.55% of the MP and 71.12% of the GP believed that the medical industry was a service industry. While 14.29% of the MP and 64.44% of the GP thought medical staff earned high salaries, 19.48% of the MP and 47.11% of the GP wanted their children to be in the medical industry. The recognition of the current status of the DPR did not affect the GP’s preference for their children’s practice (*p* < 0.05). Most MPs hoped to improve salaries (40.26%), followed by safety (17.53%) and social status (12.99%); only 8.44% of the MP wanted to improve the DPR.

**Conclusion:**

The MP’s and GP’s views on the current status of DPR, the importance of medical service attitudes, and the general sense of the medical industry were similar. However, there was a significant difference in the perception of the nature of medical services and the income of the people employed in the medical industry between the two groups. Balancing the expectations of patients in the medical industry and increasing public awareness of the actual situation in the medical industry may be a feasible way to improve the DPR.

## Background

The doctor-patient relationship (DPR) is the interactive relationship in the medical service activities between doctors and patients, as well as between the individuals and social groups that are closely related to the interests of both parties. Globally, due to the commercialization and privatization of the medical industry, the DPR has undergone tremendous changes over the past few decades [1]. DPR has changed from being doctor-centric to patient-centric. This patient-centric model of DPR reduces physician dominance, advocates greater patient control, and encourages more mutual participation. This has become the predominant model in clinical practice today [2].

In recent years, the demand for better health care in the third-world countries has gradually increased, and an expanding aging population has become a global health care problem due to limited medical resources [3]. The People’s Republic of China is a developing country with a population of about 1.4 billion. In China, medical resources are dominated by public ownership. To offer better health care, since the 2010s, the Chinese government has adopted a series of effective reforms that include expansion of the social health insurance, reform of the public hospitals, and strengthening of primary care. These reforms have succeeded in reducing mortality, increasing life expectancy, and providing better primary health care [4, 5]. However, as the aging population continues to increase, China’s limited medical resources will face huge challenges in the future [6].

From a sociological perspective, DRP depends on the ethics of both doctors and patients. From a psychological perspective, “communication behavior patterns”, “psychic distance”, “emotional quotient”, “conflict between pain relief and truth-telling”, and “body language” affect DRP [2, 7]. Studies showed that DRP is seen as the behavior and attitude of the doctor towards the patient, and the perception of the patient concerning the caring shown by the doctor [8]. A harmonious DPR can A good DPR can optimize patients’ medical experience and improve treatment efficiency, outcomes that are important to both the doctors and patients. While the DPR has been the focus of public opinion, Workplace violence experienced by doctors has been documented both in developed and developing countries, which is also in crisis in China [9]. Reports show over the past few years, violent behaviors such as violent abuse, riots, assaults and hospital protests against medical professionals have surged in China. Chinese medical professionals are being injured, disabled, or even killed by patients or their family members [10, 11]. Despite the heavy workload of Chinese medical professionals, they have been disabled, injured, or even killed by patients or their families [12]. Although there are many reports and studies on DPR in China [13–15], there are only a few in-depth and comprehensive studies have focused on improving the DPR by investigating medical personnel (MP) and the general population (GP) simultaneously. This study focused on both doctors and patients, compared the differences in perception between MP and GP on the status of DPR, medical services, and the general situation of medical work, in order to explore a feasible way to improve the DPR.

## Methods

### Study design

This was a cross-sectional study, and data were collected using questionnaires.

### Population and data collection procedure

The scope of this research survey was limited to Nanjing City, Jiangsu Province, and Zhengzhou City, Henan Province. A total of 154 MP and 329 GP respondents participated in the study. Data were collected using a self-administered online questionnaire, distributed through Questionnaire Star and WeChat apps. While participation in the study was voluntary, the participants received monetary compensation (After completing the questionnaire and submitting it successfully, each respondent will receive 10 RMB.). All participants signed the informed consent form attached to the questionnaire prior to answering any question. The participants had 15 min to answer the questionnaire.

### Questionnaire

We define DPR through three levels of “harmony”, “Tense” and “Normal”, “Harmonious” DPR mainly means that both the doctor and the patient can always complete the treatment process in a relaxed and friendly atmosphere. “Tense” DPR mainly refers to the fact that both the doctor and the patient cannot trust each other, fail to complete the medical treatment process smoothly, and cause medical disputes and even violent injuries to the doctor. “Average” refers to the state of DPR between harmony and tension. Although both doctors and patients cannot fully trust each other, there are not always medical disputes and violent incidents.

Self-administered questionnaires covering three aspects were answered by the participating MP and GP respondents. The first part of the questionnaire used by the MP included items on sociodemographic factors: Gender, Age, Job title (Primary, Intermediate, Deputy Senior, Advanced), Department (Internal Medicine, Surgical, Obstetrics and Gynecology, Pediatrics, Emergency Department, Medical Technology Department, and Other Auxiliary Departments), Years on the Job, Hospital Type (Traditional Chinese Medicine, Modern medicine and Integrated Chinese and Western Medicine), Hospital level (Community, Level 2, Level 3, Other Medical Institutions), Practice Category (Nursing, Traditional Chinese Medicine Physician, Modern Medicine Physician, Integrated Chinese, and Western Medicine Physician), Education (Technical Secondary School/College, Undergraduate, Master’s degree, Ph.D. and Above), and Place of Residence (Village/Township/Town, County-level Cities/Prefecture-level Cities, Provincial Capital Cities/Municipalities). The first part of the questionnaire used by GP included items on sociodemographic factors: Gender, Age, Place of Residence (Village/Township/Town, County-level Cities/Prefecture-level Cities, Provincial Capital cities/Municipalities), Education (Elementary School and Below, Junior and High school, Technical Secondary School/College, Undergraduate, Postgraduate and Above), Job type (Institution/Civil Service, Private Enterprise Employees, Self-employed, Unemployed and Student), and Previous Hospitalization (Yes or No).

In the second part of the questionnaire, the participants were asked whether the medical industry belonged to the service industry and whether the service attitude of medical personnel with professional skills was important. Responses from the MP and the GP cohorts were investigated separately. And investigated GP about whether the service attitude of medical staff is appropriate compared with government staff/service staff. The third part of the questionnaire was aimed at finding the difference between the MP and GP respondents’ perception of the medical industry. The MP and GP’s views on the medical staff’s income level and whether they wanted their children to be in the medical field were investigated as well. Finally, the MP’s opinion on how to improve in routine medical work (Social Status, Salary, Safety, Work time, Night Shift Frequency, Cumbersome Hospital Assessment, Doctor-patient Relationship, Working Environment) was also investigated.

### Statistical analysis

All data were incorporated into a Microsoft Excel spreadsheet. Data analyses were done using SPSS, version 22.0, and SPSSAU, version 20.0. Wilcoxon’s Sign Rank Test was implemented to compare the differences in attitude scores between the two groups. Chi-square test was used for categorical data between the groups, and categorical data were summarized using frequency distributions and percentages. A value of *p* < 0.05 was considered significant.

## Result

### Characteristics of the sample

**Fig. 1:**
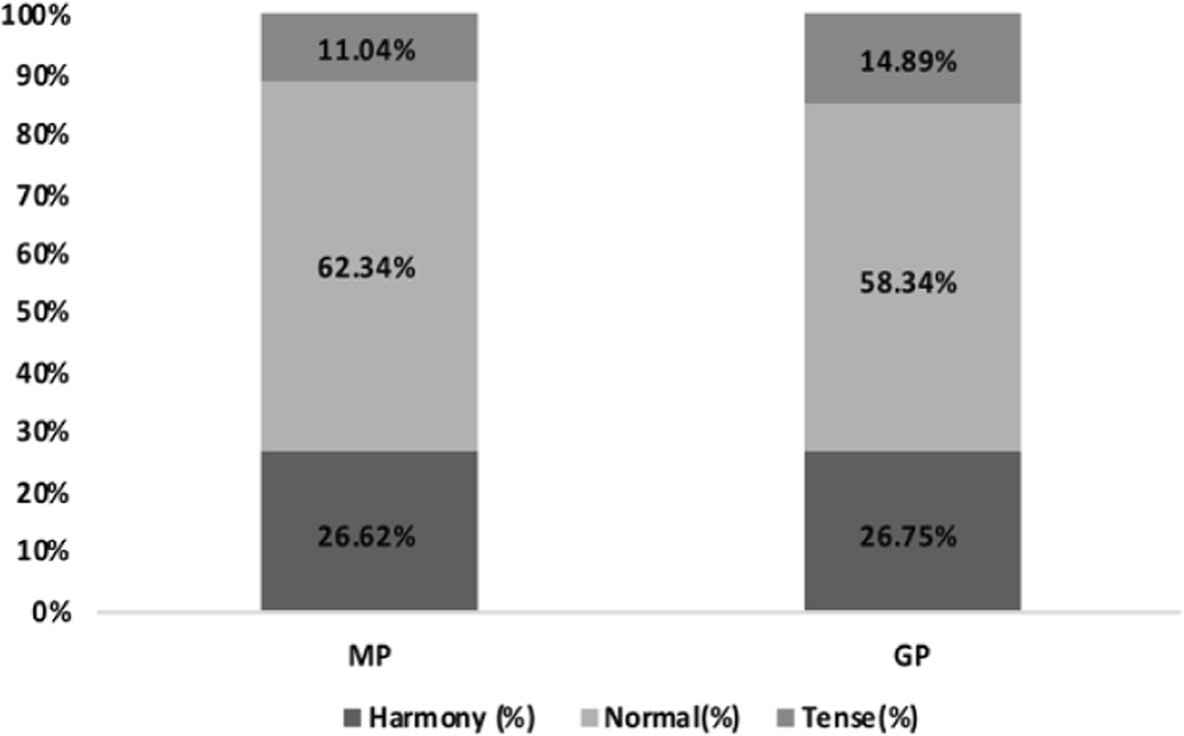
Differences between the MP and the GP in the perception of the DPR

**Fig. 2:**
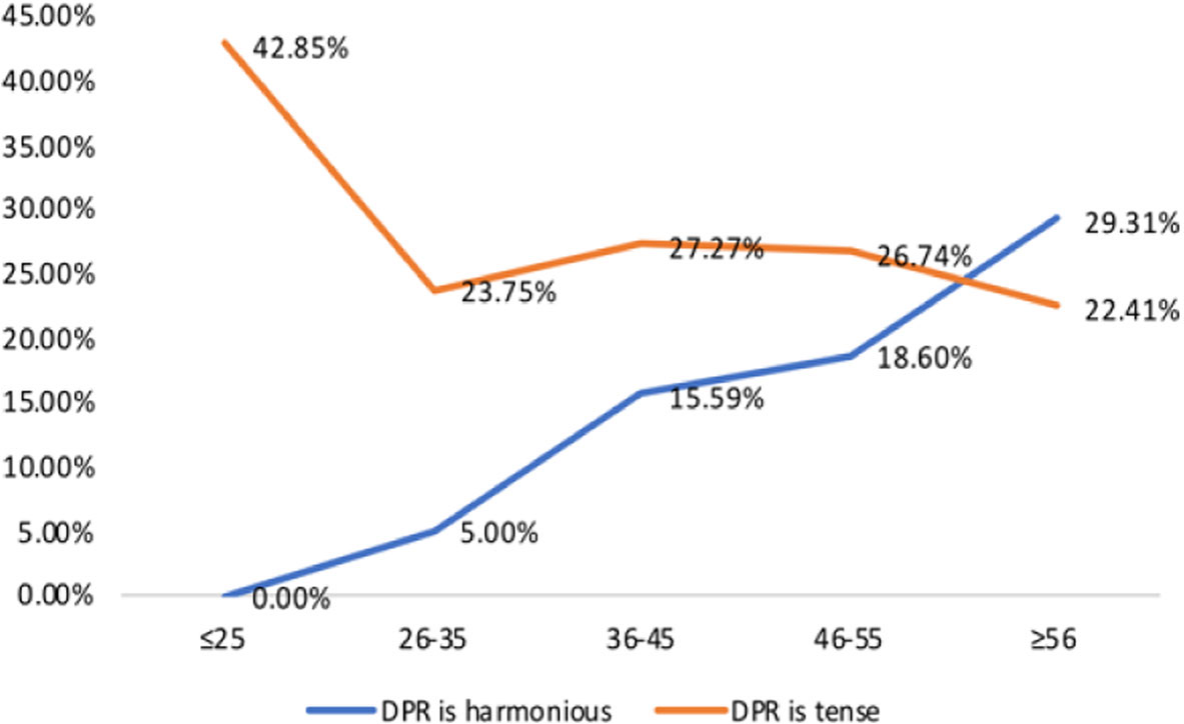
Cognition of DPR by GP of different ages

The background characteristics of the participants are shown in Table 1. Female make up the majority of participants (MP 63.64%, GP 61.40%). The largest number of Participants live in Provincial capital cities/Municipalities (MP 51.95%, GP 56.23%). The age of the participating GPs is relatively average, while the most MPs participating in the survey are 26–35 years old (53.90%). In terms of education, MPs has the most Master’s degree (50.65%) and GPs has the most Undergraduate (38.30%). A total of 41 (26.62%) of the 154 MPs believed the DPR to be tense, 96 (62.34%) thought it to be average, and only 17 (11.04%) thought it to be harmonious. Of the 329 GP respondents, 88 (26.75%) believed that the current DPR was tense, 192 (58.36%) thought it was average, and 49 (14.89%) thought it was harmonious. The cognitive difference of the current situation for the DPR between medical personnel and non-medical personnel is shown in Fig. 1. Among the MPs, age, gender, department, years on the job, professional title, hospital level, type of practice, education, and place of residence had no statistical significance on the evaluation of the DPR (*p* > 0.05). Among the GPs, gender, work type, education, place of residence, and prior hospital admittance had no statistical significance on the evaluation of the DPR (*p* > 0.05); the data are given in Table 2. Age, however, was a statistically significant factor in the GPs on the evaluation of the DPR (*p* = 0.01); the data are given in Table 3. With age, GPs tended to think DPR is harmonious, as shown in Fig. 2.

**Table 1:** Background characteristics of MPs and GPs

Group	MPs (%)	GPs (%)	P
***Gender***			0.71
Male	56(36.36)	127(38.60)	
Female	98(63.64)	202(61.40)	
***Age***			0.21
≤ 25	34(22.08)	28(8.51)	
26–35	83(53.90)	80(24.32)	
36–45	13(8.44)	77(23.40)	
46–55	22(14.29)	86(26.14)	
≥ 56	2(1.30)	58(17.63)	
***MPs’ Education***			
Technical secondary school/college	10(6.49)		
Undergraduate	57(37.01)		
Master’s degree	78(50.65)		
PhD and above	9(5.84)		
***GPs’ Education***			
Elementary school and below		5(1.52)	
Junior and high school		68(20.67)	
Technical secondary school/college		88(26.75)	
Undergraduate		126(38.30)	
Postgraduate and above		42(12.77)	
***Place of residence***			0.23
Village/township/town	21(13.64)	54(16.41)	
County-level cities/prefecture-level cities	53(34.42)	90(27.36)	
Provincial capital cities/Municipalities	80(51.95)	185(56.23)

**Table 2:** General population data of 154 MPs and the difference in the perception of the status of the doctor-patient relationship among groups

Group	Harmony (%)	Normal(%)	Tense(%)	X^**2**^	P
***Gender***				0.01	0.995
Male	6(35.29)	35(36.46)	15(36.59)		
Female	11(64.71)	61(63.54)	26(63.41)		
***Age***				3.97	0.86
≤ 25	4(23.53)	21(21.88)	9(21.95)		
26–35	7(41.18)	52(54.17)	24(58.54)		
36–45	2(11.76)	7(7.29)	4(9.76)		
46–55	4(23.53)	14(14.58)	4(9.76)		
≥ 56	0(0.00)	2(2.08)	0(0.00)		
***Department***				5.788	0.926
Internal Medicine	10(58.82)	46(47.92)	22(53.66)		
Surgical	1(5.88)	20(20.83)	9(21.95)		
Obstetrics and Gynecology	1(5.88)	3(3.13)	2(4.88)		
Pediatrics	1(5.88)	5(5.21)	2(4.88)		
Emergency department	0(0.00)	2(2.08)	0(0.00)		
Medical Technology department	0(0.00)	4(4.17)	1(2.44)		
Other auxiliary departments	4(23.53)	16(16.67)	5(12.20)		
***Years on the Job***				8.645	0.373
≤ 5	10(58.82)	69(71.88)	31(75.61)		
6–10	2(11.76)	6(6.25)	2(4.88)		
11–20	1(5.88)	2(2.08)	4(9.76)		
21–30	4(23.53)	16(16.67)	4(9.76)		
≥ 31	0(0.00)	3(3.13)	0(0.00)		
***Job title***				11.014	0.088
Primary	10(58.82)	71(73.96)	30(73.17)		
Intermediate	5(29.41)	13(13.54)	5(12.20)		
Deputy Senior	0(0.00)	6(6.25)	6(14.63)		
Advanced	2(11.76)	6(6.25)	0(0.00)		
***Hospital type***				1.715	0.788
TCM	12(70.59)	59(61.46)	24(58.54)		
MM	3(17.65)	20(20.83)	7(17.07)		
ICWM	2(11.76)	17(17.71)	10(24.39)		
***Hospital level***				3.332	0.766
Community	2(11.76)	7(7.29)	2(4.88)		
Level 2	2(11.76)	21(21.88)	8(19.51)		
Level 3	12(70.59)	56(58.33)	28(68.29)		
Other medical institutions	1(5.88)	12(12.50)	3(7.32)		
***Practice category***				8.032	0.236
Nursing	0(0.00)	4(4.17)	2(4.88)		
TCM physician	14(82.35)	68(70.83)	32(78.05)		
MM physician	1(5.88)	11(11.46)	7(17.07)		
ICWM physician	2(11.76)	13(13.54)	0(0.00)		
***Education***					
Technical secondary school/college	0(0.00)	7(7.29)	3(7.32)	9.707	0.138
Undergraduate	5(29.41)	41(42.71)	11(26.83)		
Master’s degree	9(52.94)	45(46.88)	24(58.54)		
PhD and above	3(17.65)	3(3.13)	3(7.32)		
***Place of residence***				3.38	0.496
Village/township/town	4(23.53)	11(11.46)	6(14.63)		
County-level cities/prefecture-level cities	3(17.65)	35(36.46)	15(36.59)		
Provincial capital cities/Municipalities	10(58.82)	50(52.08)	20(48.78)		

*TCM* Traditional Chinese Medicine, *MM* Modern Medicine, *ICWM* Integrated Chinese and Western Medicine Physician

**Table 3:** General population data of 329 GPs and the differences in the perception of the status of the DPR between groups

Group	Harmony (%)	Normal(%)	Tense(%)	X^**2**^	P
***Gender***				1.695	0.428
Male	23(46.94)	71(36.98)	33(37.50)		
Female	26(53.06)	121(63.02)	55(62.50)		
***Age***				25.26	0.001
≤ 25	0(0.00)	16(8.33)	12(13.64)		
26–35	4(8.16)	57(29.69)	19(21.59)		
36–45	12(24.49)	44(22.92)	21(23.86)		
46–55	16(32.65)	47(24.48)	23(26.14)		
≥ 56	17(34.69)	28(14.58)	13(14.77)		
***Job type***				15.394	0.052
Civil Service	16(32.65)	70(36.46)	20(22.73)		
Private enterprise employees	17(34.69)	70(36.46)	42(47.73)		
Self-employed	6(12.24)	21(10.94)	7(7.95)		
Unemployed	10(20.41)	21(10.94)	10(11.36)		
Student	0(0.00)	10(5.21)	9(10.23)		
***Education***				13.57	0.094
Elementary school and below	3(6.12)	1(0.52)	1(1.14)		
Junior and high school	10(20.41)	40(20.83)	18(20.45)		
Technical secondary school/college	16(32.65)	45(23.44)	27(30.68)		
Undergraduate	17(34.69)	80(41.67)	29(32.95)		
Postgraduate and above	3(6.12)	26(13.54)	13(14.77)		
***Place of residence***				5.188	0.269
Village/township/town	8(16.33)	32(16.67)	14(15.91)		
County-level cities/prefecture-level cities	9(18.37)	50(26.04)	31(35.23)		
Provincial capital cities/Municipalities	32(65.31)	110(57.29)	43(48.86)		
***Previous Hospitalization***				4.217	0.121
Yes	47(95.92)	179(93.23)	87(98.86)		
No	2(4.08)	13(6.77)	1(1.14)

### Differences between the MP’s and the GP’s perception of medical services

**Fig. 3:**
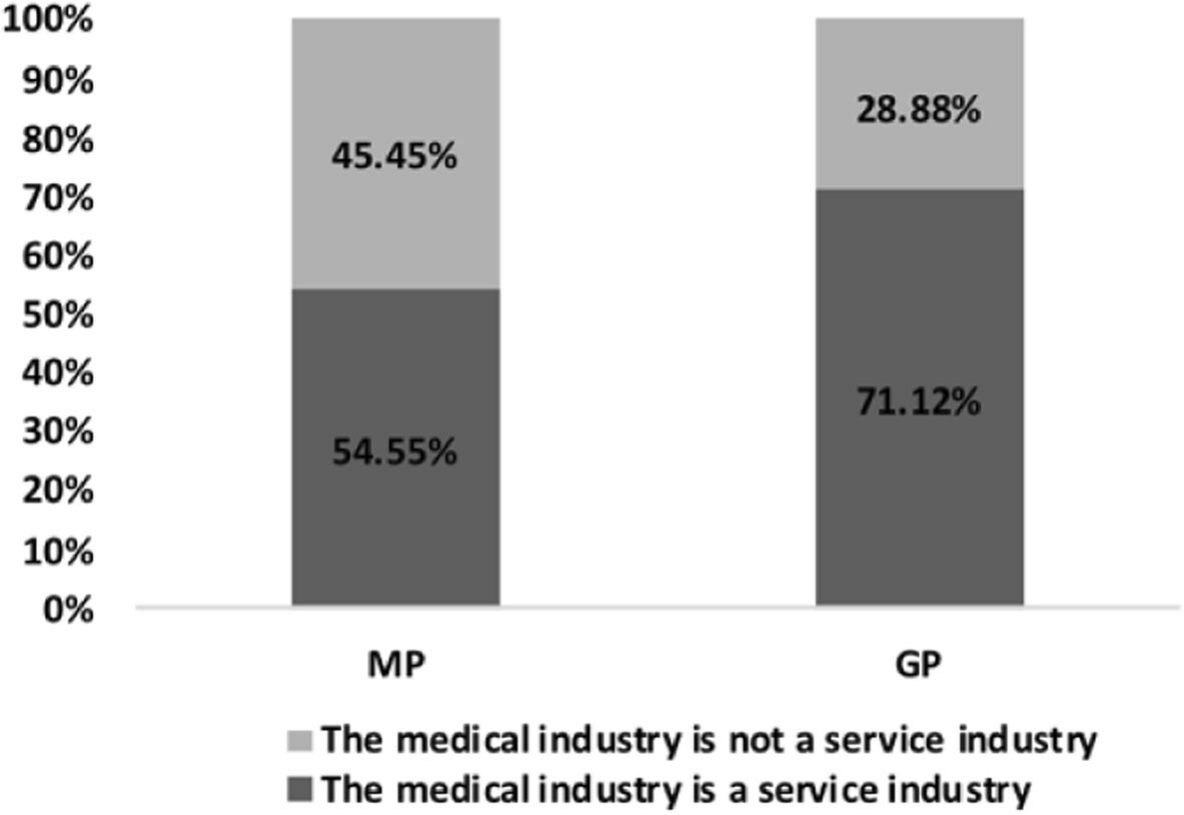
Differences in perception between the MP and the GP respondents on medical services

**Fig. 4:**
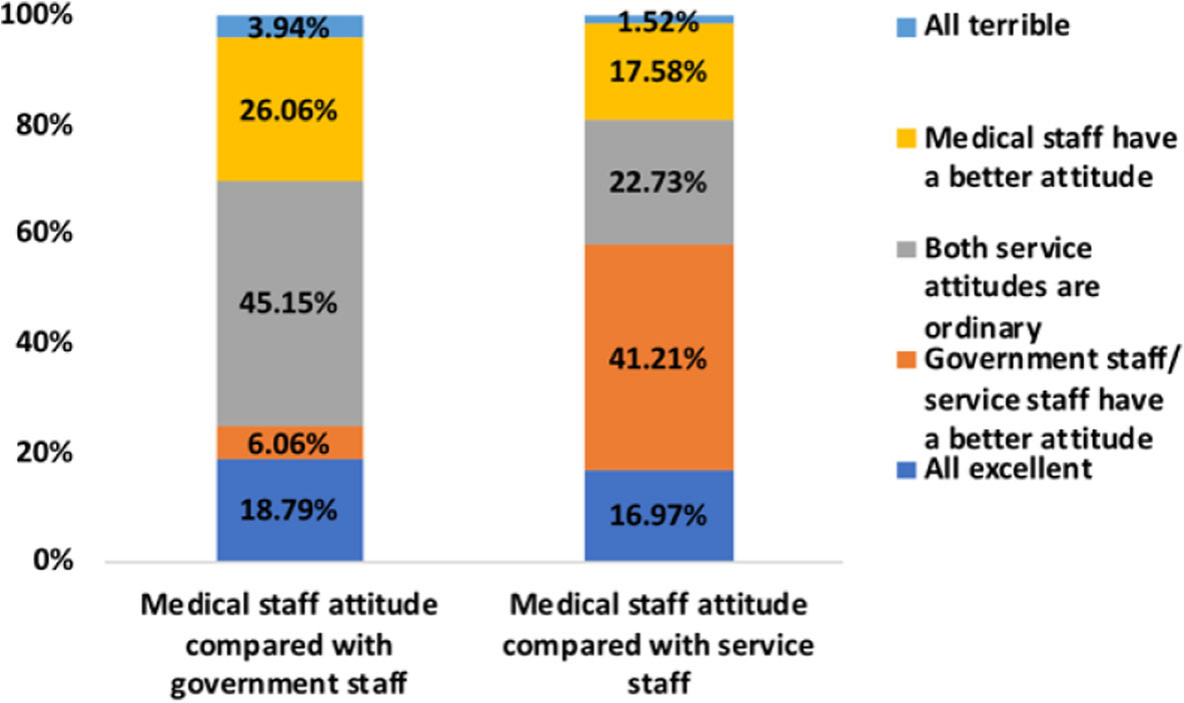
The GPs’ perception of the difference in service attitude between medical staff and government staff/service staff

A total of 84 (54.55%) of the 154 MPs believed that the medical industry was a service industry, and 70 (45.45%) thought it was not. A total of 150 (97.4%) MPs thought that the service attitude of medical staff was as important as professional skills, and only 4 MPs (2.6%) considered it was not. While 234 (71.12%) of the 329 GPs thought that the medical industry was a service industry, 95 (28.88%) did not. Interestingly, 315 (95.74%) GPs thought that the attitude of medical staff is more important than professional skills and only 14 GPs (4.26%) considered it to be unimportant. The difference between MP and GP’s perception of medical services is shown in Fig. 3. Age, gender, department, years on the job, job title, hospital level, practice category, education and place of residence had no statistical significance for the MP’s perception on whether the medical industry was a service industry (*p* > 0.05); the data are shown in Table 4. Gender and prior hospitalization were statistically significant factors for the GPs’ perception of whether the medical industry was a service industry. Education has statistical significance for GPs’ perception of the importance of the service attitude of medical staff (*p* < 0.05); the data are given in Table 5. The GPs’ perception difference in service attitudes between medical staff and government staff/service staff is shown in Fig. 4. Comparison of the service attitudes between the medical staff and the government staff shows that 45.15% of the GPs believed that the service attitude of both sectors was good in general, while 26.06% believed the service of the medical staff was better, and only 6.06% thought service attitude was bad in both sectors. A comparison of the service attitudes between the medical staff and the service staff showed that 41.25% of the GPs believed that the service attitude of the service staff was better, 17.58% thought that the medical staff was better, and only 1.52% thought both were bad.

**Table 4:** Differences in the MPs’ perception of medical services

Is the medical industry a service industry?			
Group		X^**2**^	P
Years on the Job	≤5/6–10/11–20/21–30/≥31	10.413	0.034
Hospital type	TCM/MM/ICWM	8.286	0.016

**Table 5:** Differences in the GPs’ perception of medical services

(a) Compared with professional skills, is the service attitude of medical staff important?			
**Group**		**X**^**2**^	**P**
Gender	Male/Female	6.684	0.010
Previous Hospitalization	Yes/No	30.08	0.000
(b) Is the medical industry a service industry?			
**Group**		**X**^**2**^	**P**
Education	Elementary school and below, Junior and high school, Technical secondary school/college, Undergraduate, Postgraduate and above	20.807	0.000

### Differences in perception between the MP and the GP on the medical industry

#### Differences in perception between the MP and the GP on medical industry income

**Fig. 5:**
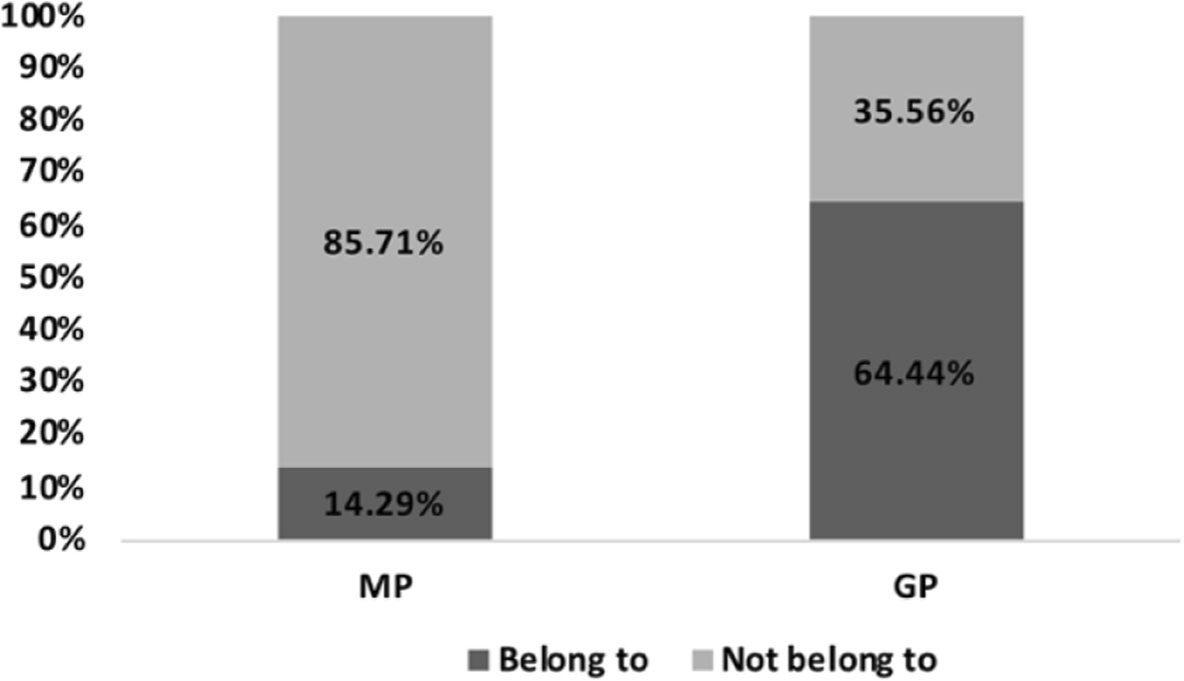
Differences in perception between the MP and the GP respondents on medical staff income level

Of the 154 MP respondents, 132 (85.71%) thought that medical staff did not earn high salaries, and only 22 (14.29%) thought they did. All factors such as age, gender, department, years on the job, job title, hospital level, practice category, education, and place of residence had no statistical significance for MP’s cognition of the medical staff’s income level, (*P* > 0.05). Of the 329 GP respondents, 117 (35.56%) thought that medical staff did not earn high salaries, and 212 (64.44%) thought they did. Two factors, age and job type, have statistical significance for MP’s cognition of the medical staff’s income level (*P* < 0.05); the data are given in Table 6. The difference between the MP and GP respondents’ perception of the medical staff’s income level is shown in Fig. 5.

**Table 6:** Differences in GPs’ perceptions of income from the medical industry

Group		X^**2**^	P
Age	≤25/26–35/36–45/46–55/≥56	10.874	0.028
Job type	Civil Service, Private enterprise employees, Self-employed, Unemployed, Student	10.213	0.037

#### Differences between MP and GP respondents in choosing whether their children are engaged in the medical industry

**Fig. 6:**
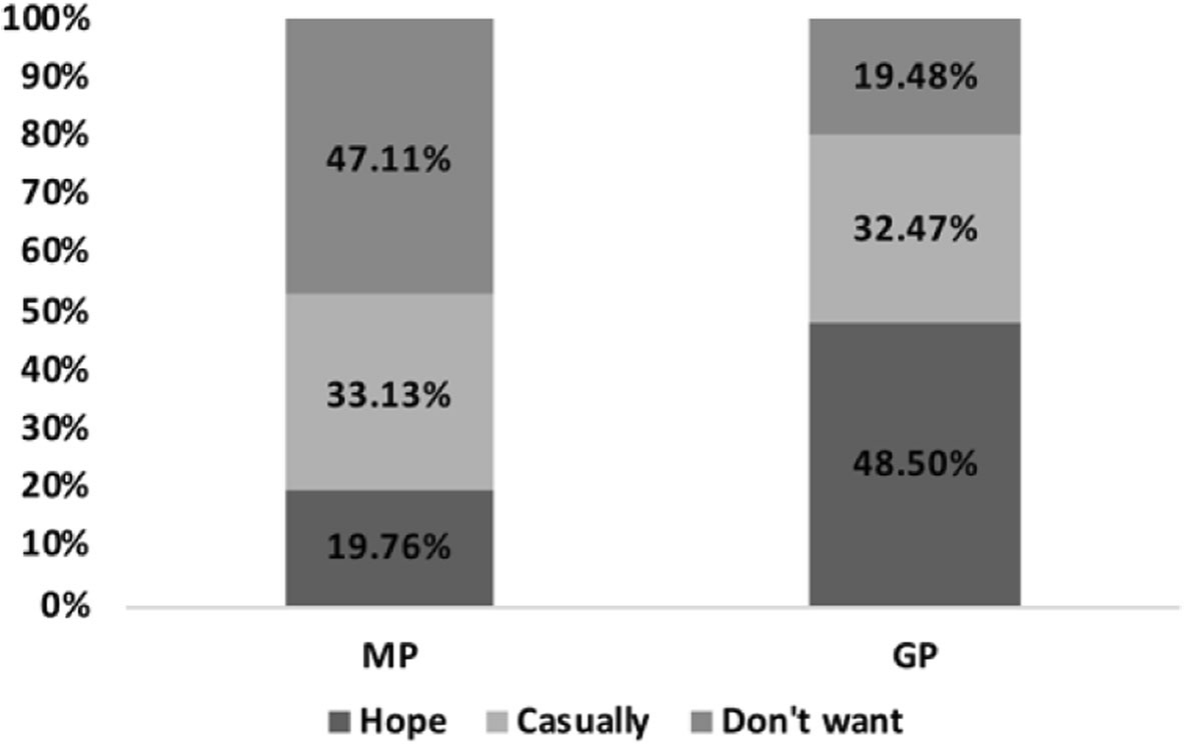
Differences between the MP and the GP on whether their children should be in the medical industry

**Fig. 7:**
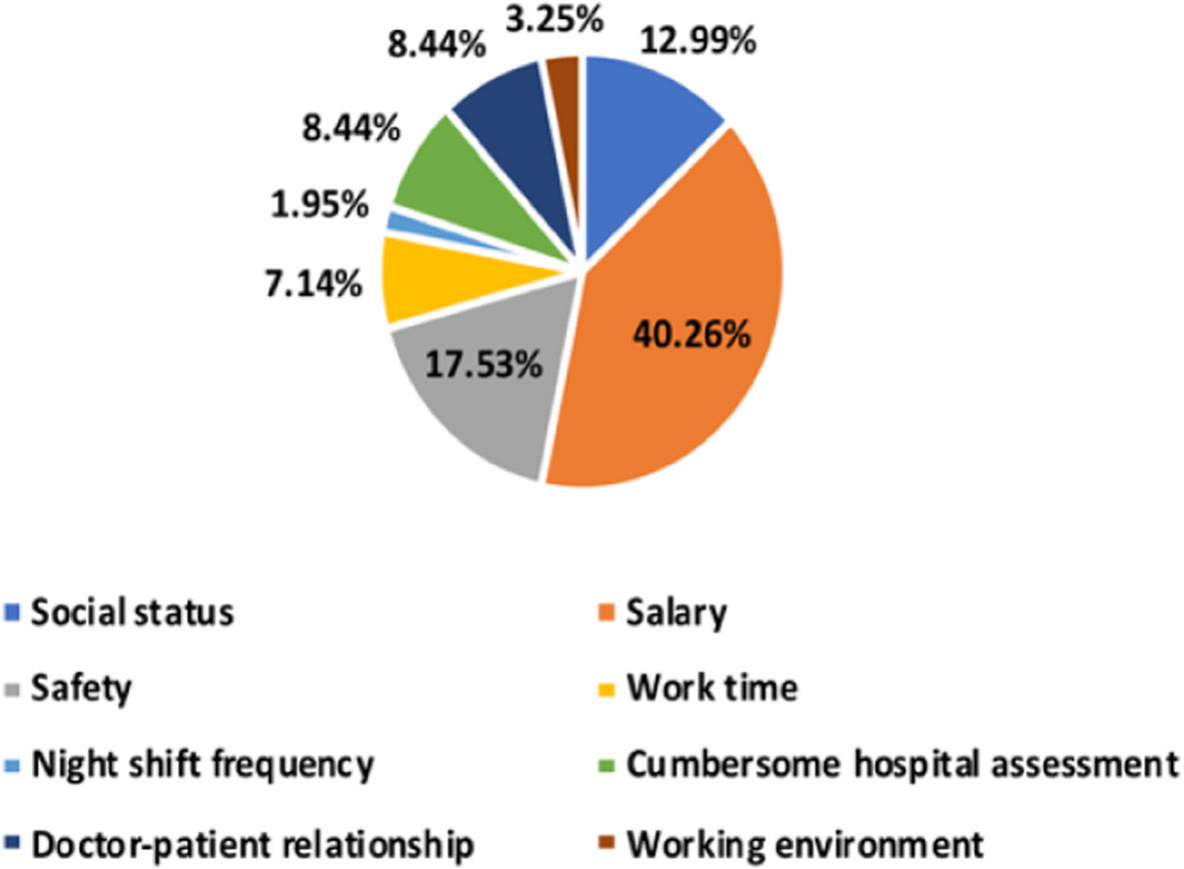
MP’s choice of what they want to improve in routine medical work

Of the 154 MP respondents, 74 (48.05%) did not want their children to be in the medical industry, and only 30 (19.48%) wanted that. In contrast, 155 (47.11%) of the 329 GP respondents wanted their children to be in the medical profession, and only 65 (19.76%) did not. The difference between the two in choosing whether they preferred their children to be in the medical industry is shown in Fig. 6. Several factors, such as job title, education, perception of medical staff’s income, and what MPs prioritized to improve in routine medical work, were statistically significant for the MPs to choose whether they wanted their children to be in the medical industry (*p* < 0.05); the data are shown in Table 7. Age, job type, cognition of medical staff’s income level, perception differences in the DPR were statistically significant factors for the GP to choose whether they wanted their children to be in the medical industry (p < 0.05); the data are given in Table 8. Among the 154 MPs, the most wanted to improve their salary (40.26%), followed by safety issues (17.53%); the improvement of the working environment was the least preferred factor (3.25%), as shown in Fig. 7.

**Table 7:** Differences affecting MP’s choice of whether their children are engaged in the medical industry

Group		X^**2**^	P
Job title	Primary, Intermediate, Deputy Senior, Advanced	13.032	0.043
Education	Elementary school and below, Junior and high school, Technical secondary school/college, Undergraduate, Postgraduate and above	13.301	0.038
Whether medical staff belong to high-income groups.	Belong to/not belong to	12.318	0.002
MP’s choice of what they want to improve in routine medical work.	Social status, Salary, Safety, Work time, Night shift frequency, Cumbersome hospital assessment, Doctor-patient relationship, Working environment	34.86	0.002

**Table 8:** Differences affecting the GP’s choice of whether their children should be in the medical industry

Group		X^**2**^	P
Age	≤25/26–35/36–45/46–55/≥56	19.371	0.013
Job type	Institution/Civil Service, Private enterprise employees, Self-employed, Unemployed, Student	25.104	0.001
Whether medical staff belong to high-income groups.	Belong to/not belong to	9.949	0.007
Current status of DPR.	Harmony /Normal/Tense	13.869	0.008

## Discussion

Economic development and an increase in the aging population have heightened the demand for medical resources [16]. While China is in a period of rapid economic development, her large population, the polarization of the rich and the poor, the increase in the aging population, and the uneven distribution of medical resources have created complex problems in China’s medical industry [17, 18]. Rational allocation of medical resources and finding ways to improve treatment efficiency are two of the most pressing issues right now. Finding a way to increase the DPR, in this context, can play an important role in reforming China’s health care system. Since the medical industry closely related to people’s wellbeing, improved DPR would be mutually beneficial to both the doctors and the patients. For example, improved DPR can improve treatment efficacy and better medical experience to the patients, while it will provide the doctors with better working environments and increase work efficiency. Although the Chinese government has taken many measures to improve the DPR, success has been minimal. Our data on MP and GP respondents’ perception of the DPR showed that only 11.04% of the MPs and 14.89% of the GPs believed that the current DPR is harmonious. This indicates that the DPR is at a relatively tense level in China, which is consistent with previous research [10]. However, there was a significant difference in the perception of the nature of medical services and the income of the people employed in the medical industry between the two groups.

### Analysis of the status of the DPR between the MP and the GP and the difference in perception about medical service

The service industry is considered to be service-oriented, such as restaurant waiters and salespeople. Whether the medical industry is regarded as service-oriented or business oriented, it has an impression of patients’ expectations of MP, and in turn affects DPR. A significant percentage of the participants, 97.4% of the MP and 95.74% of the GP respondents, believed that the service attitude of medical staff was as important as the MPs’ professional skills. Moreover, 54.55% of the MPs believed that the medical industry was a service industry, and 71.12% of the GPs believed that the medical industry was a service industry. These data showed that while the MP and the GP had a similar understanding of the importance of service attitude in the medical industry, the two groups’ opinions differed when it came to the nature of the service of the medical industry. The GP group tended to think that the medical industry was service-oriented, while the MP group thought the opposite was true. This may lead to differences in the expectations and communication methods between doctors and patients during medical treatment, which exacerbates the DPR crisis [19]. A comparison of the service attitude between medical staff with government staff showed that 26.06% of the GPs thought that medical staff offered better service, and only 6.06% thought that the government staff offered better service. Compared with traditional service industries, 17.58% of the GPs thought that medical staff offered service, and 41.25% thought that the reverse was true. This indicated that the service attitude of medical staff between that of government staff and staff of traditional service industries is significantly better than the non-traditional service industry. This suggested that to a certain extent, the service attitude of medical staff is not directly responsible for the tension in the DPR.

### Analysis of the difference between MP and GP respondents’ general perception of the medical industry

Our data show that 85.71% of the MPs thought that they did not earn high salaries while 14.29% thought they did. Among the GP respondents, 35.56% thought that MP did not earn high salaries while 212 (64.44%) thought they did. These data showed that there was a difference in perception on MPs’ income between the MP and the GPs: the majority of the GP thought the MPs’ income was high while the reverse was true for the MP. While 47.11% of the GPs wanted their children to be in the medical profession, only 19.48% of the MPs wanted that. Age, job type, cognition of medical staff’s income level, and the status quo of doctor-patient relationship differences had an impact on whether GP wanted their children to choose the medical profession. Interestingly, the recognition of the current status of the doctor-patient relationship did not affect GP’s choice in this regard. Among the GP who wanted their children to be in the medical industry, 83.84% thought the DPR was currently tense, and only 14.89% thought the DPR was harmonious. Our data on the MPs’ preferred aspect to improve the routine medical works showed that majority of the MPs hoped to improve their salaries (40.26%), followed by safety (17.53%) and social status (12.99%); only 8.44% wanted to improve the DPR. Our data was consistent with the results of another study [20]. These results suggested that the GPs’ perception of the medical industry does not change with either tense or harmonious DPR. The factors medical practitioners need to improve most are related to personal life, such as salary, safety, and social status. The problems in the DPR is one of the common problems of both developed and developing countries [21], which has also intensified in China. The general public believes this happens because the patients do not understand the medical staff’s instructions and do not pay attention to the instructions. Thus, the media always tends to portray doctors and nurses as heroes. Headlines such as “Sick doctor goes to work”, “Writing medical orders while dripping”, “Working continuously for 48 hours during the Spring Festival” encourages doctors to sacrifice themselves for patients [13, 22]. Continuously raising the moral standards of the medical industry and raising the professional positioning of the medical industry while ignoring the needs of medical personnel as ordinary people will inevitably increase their professional burden as patients will have unreasonable expectations from the medical staff. Studies have shown that violent medical injuries mostly occur in areas where high-quality medical resources are concentrated, such as top hospitals, and this mostly happens because patients expect too much from the doctors [11, 23]. Whether the MP and the GP respondents’ different perceptions about the nature of services in the medical industry is related to high demands and ethical standards set by public opinion needs to be investigated in the future.

## Conclusions

This study indicated that the MP’s and the GP’s perception of the current status of DPR, the importance of medical service attitudes, and the general sense of the medical industry are similar. Both groups believed that DPR is currently tense and affirmed the importance of service attitude in the medical industry. Their main difference lies in the perception of the nature of medical services and the income of the medical industry. Unlike the MPs, the GPs believed that the medical industry belongs to the service industry and that medical personnel belonged to the high-income group. The solution to the DPR crisis lies in addressing the problem from both the medical professionals’ and patients’ perspectives, finding the key discrepancies, and implementing measures to minimize those discrepancies. Balancing the expectations of patients in the medical industry and increasing public awareness of the real situation in the medical industry may be a feasible way to improve the DPR.

## Limitations and suggestions for future research

We surveyed MP and GP from Jiangsu and Henan provinces. The sample size of this study is not large enough. This makes our research might not represent the whole situation in China and unable to fully reflect the views of people from rural areas on the DPR. In our future research, we will expand the scope of the survey in order to more comprehensively reflect the views on DPR of Chinese people, and the difference between people’s demand for medical services in developed and underdeveloped regions.

## Data Availability

All data supporting the study is presented in the manuscript or available upon request from the corresponding author of this manuscript (Hongguang Zhou) at Email: 260105@njucm.edu.cn
